# Lupus mesenteric vasculitis disguised as urticaria with abdominal pain: a case report with 10 months follow-up observation

**DOI:** 10.3389/fimmu.2025.1679036

**Published:** 2025-10-29

**Authors:** Chaoyuan Huang, Jiahao Mo, Zhenhao Ye

**Affiliations:** ^1^ Department of Gastroenterology, The Second Affiliated Hospital of Guangzhou University of Chinese Medicine, Guangzhou, China; ^2^ Second Clinical Medical College, Guangzhou University of Chinese Medicine, Guangzhou, Guangzhou, China

**Keywords:** systemic lupus erythematosus, mesenteric vasculitis, glucocorticoid, nonspecific abdominal pain, case report

## Abstract

Systemic Lupus Erythematosus (SLE) is a complex autoimmune disease characterized by multi-system involvement, with gastrointestinal manifestations often presenting diagnostic challenges due to their non-specificity. Mesenteric vasculitis, a rare but severe complication of SLE, carries a high misdiagnosis rate and mortality if it cannot be identified promptly. We report a case of a 27-year-old female with SLE presenting initially with atypical abdominal pain and duodenal edema. Due to her history of urticaria, she was initially mistaken for urticaria related abdominal pain. After a correct diagnosis, there was still progress despite conventional glucocorticoid therapy. The patient achieved remission following high-dose methylprednisolone pulse therapy, with sustained stability over 10 months of follow-up. This highlights a focus on long-term efficacy with extended follow-up, which is less common in similar past reports. This case highlights the importance of early recognition of SLE-related mesenteric vasculitis, particularly in patients with non-specific gastrointestinal symptoms, and emphasizes the necessity of aggressive immunosuppressive therapy to improve prognosis.

## Introduction

Systemic Lupus Erythematosus (SLE) is a chronic autoimmune disorder characterized by widespread inflammation and multi-organ involvement. Lupus Mesenteric Vasculitis (LMV) is a rare but severe complication of SLE, often presents with non-specific symptoms such as abdominal pain, diarrhea, and ascites, leading to a misdiagnosis rate as high as 72.7% at initial presentation (1, 2). Delayed or inadequate treatment significantly increases mortality, which may exceed 50% in severe cases (3). Early recognition and prompt initiation of appropriate treatment are critical to preventing disease progression. This report describes a case of SLE with mesenteric vasculitis presenting with atypical abdominal pain, which responded poorly to standard glucocorticoid therapy but improved dramatically with high-dose pulse therapy, underscoring the challenges in diagnosis and management of this rare complication.

## Case presentation

**Figure 1:**
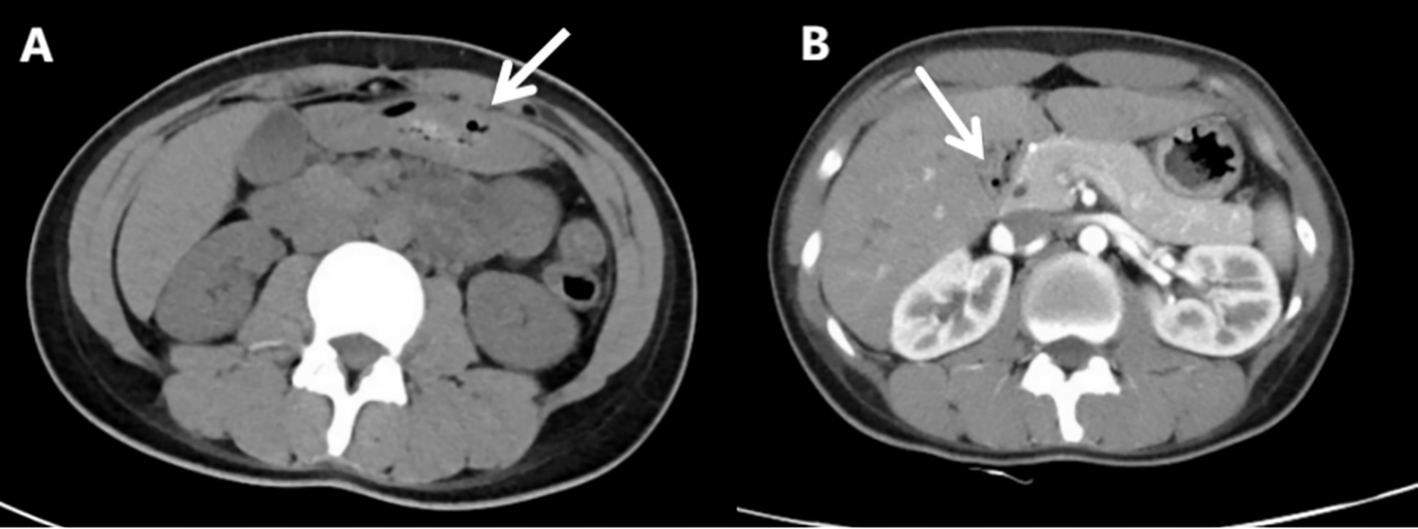
Abdominal computed tomography scan(Aug 28^th^, 2024). **(A)** The arrangement of the jejunum in the left upper abdomen is slightly disordered, with thickening of the intestinal wall. **(B)** Mild submucosal edema in the horizontal segment of the duodenum.

A 27-year-old female with a history of urticaria and tattooing presented to the emergency department with severe upper abdominal colic, which worsened progressively over 4 days following several episodes of watery diarrhea. Initial laboratory tests showed decreased total protein (62.5 g/L) and albumin (31.5 g/L), with mild acid-base and metabolic disturbances. Blood cell counts, serum amylase, lipase, and β-HCG were normal, ruling out pancreatitis, gynecological emergencies, and infectious causes. Abdominal CT revealed slightly disorganized jejunal loops with wall thickening and mild submucosal edema in the horizontal segment of the duodenum (**Figures 1A, B**). A provisional diagnosis of acute gastroenteritis was made, and the patient received analgesics, proton pump inhibitors, and prophylactic antibiotics, but her pain worsened.

**Figure 2:**
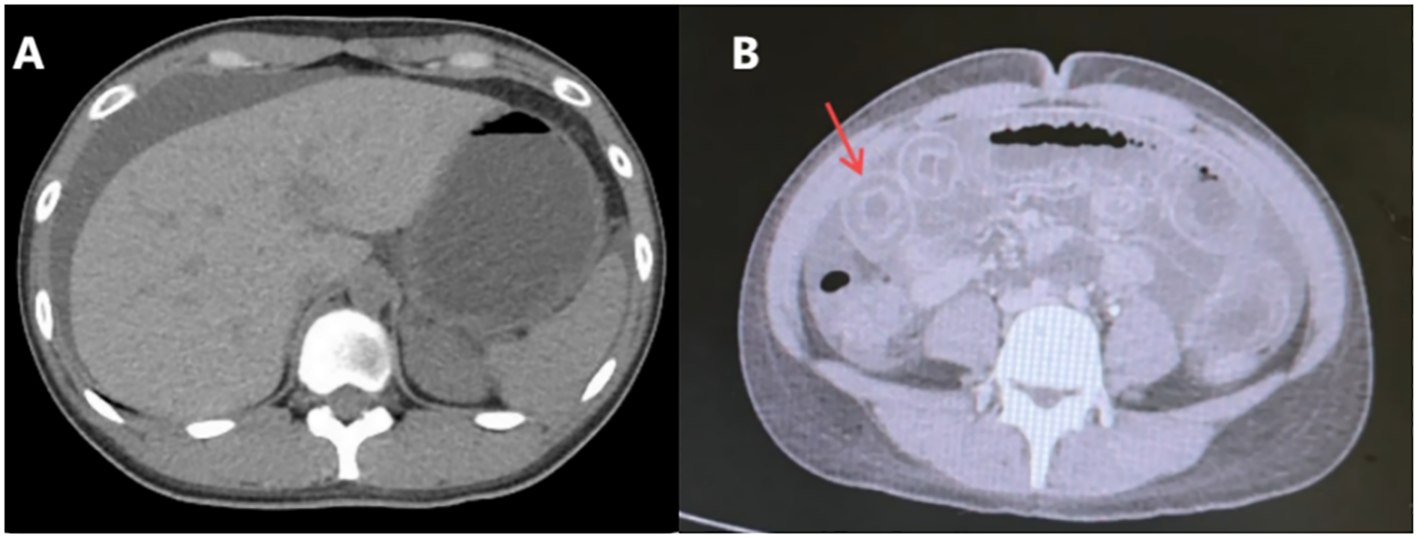
Re-examination of abdominal computed tomography scan(Aug 31^st^, 2024). **(A)** Moderate ascites. **(B)** Widespread edema and thickening of the intestinal wall in the horizontal segment of the duodenum, the entire segment of the jejunum, and the proximal segment of the ileum (target ring sign).

After admission to the Department of Gastroenterology, further workup revealed decreased complement C3 (0.29 g/L) and C4 (0.05 g/L), elevated total IgE (161.80 IU/mL), and positive autoantibodies including ANA, anti-histone, anti-nucleosome, anti-Ro-52, anti-SSA, anti-ssDNA, and anti-dsDNA antibodies. These findings supported systemic autoimmunity rather than isolated urticarial vasculitis. Reviewing her past history, it was found that she had urticaria attack one month ago. Reexamination CT showed that there was a moderate amount of ascites (**Figure 2A**), and her horizontal duodenum, jejunum and proximal ileum showed extensive edema and thickening (target ring sign) (**Figure 2B**).

**Figure 3:**
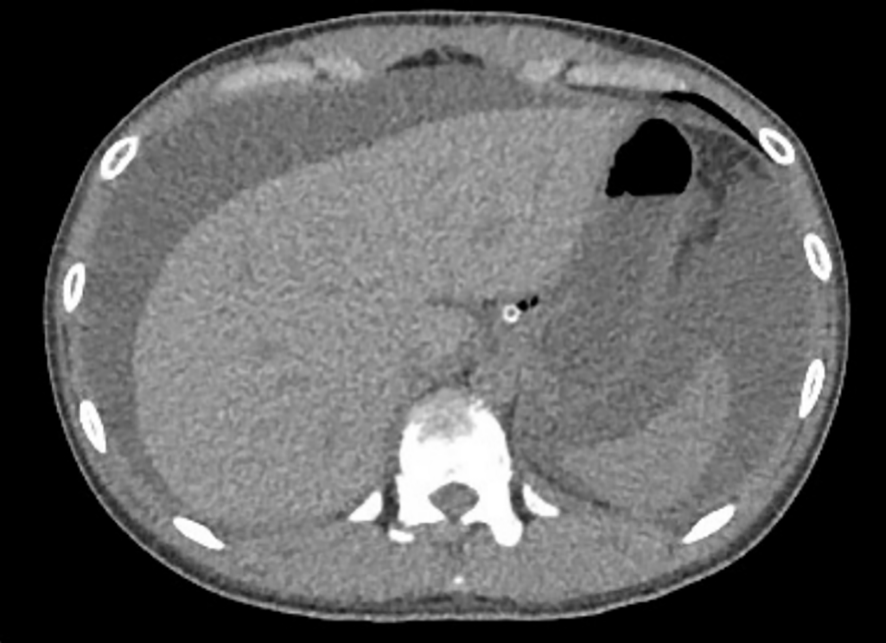
Re-examination of abdominal computed tomography scan (Sept 1^st^, 2024). A large amount of ascites, which has increased compared to before. Widespread thickening and edema of the abdominal intestinal wall.

Based on the patient’s history of urticaria, abdominal urticaria was initially considered at that time. We used the hormone methylprednisolone succinate sodium (40mg qd) for anti-inflammatory and immune suppression, in combination with diphenhydramine to suppress immune related diseases in urticaria. However, abdominal pain persisted. CT re-examination results showed a large amount of ascites (**Figure 3**), indicating rapid disease progression. Besides, pleural effusion was present bilaterally. We increased the dosage of methylprednisolone sodium succinate to 120mg/day. On the same day, 1900ml of pale red bloody ascites was drained (exudate, without bacteria or clots). Analysis of the ascites revealed total protein 37.9 g/L, and concurrent serum total protein was 54.3 g/L, resulting in a Serum-Ascites Albumin Gradient (SAAG) of 16.4 g/L and an ascites-to-serum protein ratio of 30.2%. Since an SAAG >11 g/L indicates transudative ascites (typically associated with non-inflammatory causes such as cirrhosis, rather than inflammatory conditions like tuberculous peritonitis), this patient’s ascites was classified as transudative. Follow-up CT showed increased ascites, worsening intestinal edema, and dilatation with gas accumulation. Based on the 2019 EULAR/ACR Classification Criteria for SLE (**Supplementary Figure S1**), the patient scored 15 points (fulfilling the entry criterion of positive ANA and additive criteria including hematologic abnormalities, low complement, and SLE-specific antibodies). We considered the diagnosis of SLE. The patient was transferred to the Department of Rheumatology for specialized SLE management. But on rheumatology examination, no classic SLE stigmata (malar rash or discoid lesions) were noted, and severe epigastric pain and abdominal distension persisted. Liver function tests showed elevated alanine transaminase (50 U/L) and decreased total protein (43.8 g/L). Treatment was escalated to 20% human albumin (10 g/day) for hypoalbuminemia, followed by high-dose methylprednisolone pulse therapy (500 mg/day for 3 days) with supportive care (hepatoprotection, antibiotics, electrolyte correction).

Three days later, abdominal pain improved significantly. Over the next week, methylprednisolone was tapered from 120 mg/day to 80 mg/day, in combination with intravenous cyclophosphamide (0.4g as a single pulse). To clarify the sequence of clinical events and laboratory changes, a timeline of key parameters across critical follow-up time points was provided (**Table 1**).

**Table 1:** Timeline of events and laboratory parameters during the clinical course.

Variables	August,30 2024	September,1 2024	September,8 2024	February,17 2025	June,19 2025	Reference range
Events	upper abdominal colic and watery diarrhea stage	after first use of methylprednisolone	after using high-dose methylprednisolone shock therapy	first follow-up after treatment	second follow-up after treatment	/
White blood cell count (×10^9^/L)	6.1	18.87	7.77	6.58		3.50–9.50
Hemoglobin (g/L)	137	151	124	152		115-150
Platelet (×10^9^/L)	269	292	305	332		125–350
Lymphocyte count (×10^9^/L)	0.76	2.06	1.46	1.77		1.10-3.20
Eosinophil count(×10^9^/L)	0.01	0.00	0.25	0.01		0.02-0.52
Urine protein (/HP)		Negative (−)		1+		Negative (−)
Urine microalbumin (mg/L)		8.80				0-30
Hypersensitive C-Reactive protein (mg/L)	1.15	5.22				0.5-10
Procalcitonin(ng/ml)	0.04	0.08				0.00-0.05
Lactate Dehydrogenase(U/L)		318				0-130
Albumin(g/L)	35.3	30	25.8	44.1	43.1	40-55
Creatinine(μmol/L)	72	72	60	80		41-73
D-dimer (mg/L FEU)	10.64	5.04				0.00-0.50
Fibrinogen Degradation Product(mg/L)	23.72	11.95				
Lipase (U/L)	56	22				23-300
Amylase (U/L)	101	64				35-135
Erythrocyte sedimentation rate (mm/h)	26			13		0-32
Rheumatologic and immunologic markers						
Total immunoglobulin E (IU/mL)	161.8					0–100
Immunoglobulin A (g/L)	2.08					1.00–4.20
Immunoglobulin G (g/L)	17.8			8.66		8.60–17.40
Immunoglobulin M (g/L)	0.9			0.79		0.50–2.80
Complement C3 (g/L)	0.29			0.93		0.7–1.4
Complement C4 (g/L)	0.05			0.23		0.1–0.4
Antinuclear Antibody	Positive (+)					Negative (−)
Anti-Ro-52 antibody	Strong positive (+++)					Negative (−)
Anti-SSA antibody	Strong positive (+++)					Negative (−)
Anti-dsDNA antibody	Positive (+)					Negative (−)
Anti-Smith antibody	Negative (−)					Negative (−)
Tumor markers						
CEA (ng/L)	0.58					0.00–5.00
AFP (ng/L)	1.96					0.00–7.00

After discharge, the patient was prescribed oral prednisolone (50 mg/day), alongside mycophenolate mofetil (0.75 g twice daily) and hydroxychloroquine (200 mg daily). Prednisolone was tapered by 5 mg weekly to a maintenance dose of 15 mg/day. She completed a 3-month course of cyclophosphamide with a total cumulative dose of 3 g. In April 2025, belimumab (480 mg monthly) was added to the regimen to further control subclinical immunologic activity. By late May 2025, mycophenolate mofetil was discontinued and replaced with cyclosporine (100 mg daily) as part of a tailored maintenance strategy aimed at minimizing glucocorticoid dependence and preventing relapse. The treatment regimen at this stage consisted of prednisolone (5 mg/day), cyclosporine (100 mg/day), belimumab (monthly), and hydroxychloroquine (200 mg/day). Hydroxychloroquine was discontinued in July 2025. From discharge in October 2024 to August 2025, the patient remained clinically stable with no recurrence of symptoms.

## Discussion

Mesenteric vasculitis is a rare but severe complication of SLE, often presenting with non-specific gastrointestinal symptoms (2). In this case, based on the patient’s history of urticaria, LMV was easily misdiagnosed as urticaria related abdominal pain, leading to a delayed diagnosis. The initial presentation of diarrhea and abdominal pain, combined with CT findings of intestinal wall thickening, initially suggested acute gastroenteritis. However, persistent symptoms despite antibiotics, combined with subsequent detection of ascites, hypocomplementemia, and positive autoantibodies, prompted reconsideration of SLE. It was validated by the patient’s 15-point score on the 2019 EULAR/ACR criteria, which provided objective confirmation of SLE. The timeline of events and laboratory parameters (**Table 1**) further clarifies disease progression: for example, the increase in white blood cell count (18.87×10^9^/L on September 1) reflected ongoing inflammation, while the gradual normalization of complement C3/C4 (from 0.29/0.05 g/L to 0.93/0.23 g/L by September,8 2024) and albumin (from 30 g/L to 43.1 g/L by June,19 2025) correlated with therapeutic response. Renal involvement was minimal. Notably, the patient had no history of drug exposure or other conditions known to induce mesenteric vasculitis.

Key differential diagnoses for mesenteric vasculitis include other systemic vasculitides (e.g., ANCA-associated vasculitis), inflammatory bowel disease, infectious enterocolitis, ischemic bowel disease, and neoplasia. These were excluded based on the absence of typical clinical, serological, or imaging features: no perinuclear or cytoplasmic ANCA was detected; stool cultures and Clostridium difficile toxin were negative; and tumor markers and imaging showed no evidence of malignancy. Further, she had no fever, and inflammatory markers (e.g., white blood cell count, procalcitonin) remained within normal ranges, which are inconsistent with active infection. Ischemic bowel disease was unlikely given that this condition predominantly affects elderly patients and classically presents with acute abdominal pain accompanied by hematochezia, none of which were observed in this young female patient. The patient’s rapid clinical response to immunosuppressive therapy further supported an autoimmune (rather than infectious, ischemic, or neoplastic) etiology, confirming the diagnosis of lupus mesenteric vasculitis.

The diagnostic challenges in this case highlight the importance of a high index of suspicion for SLE in young patients with unexplained abdominal pain, especially when accompanied by serological abnormalities (such as positive ANA, anti-dsDNA, hypocomplementemia). The presence of “target sign” on CT, indicative of intestinal wall edema and inflammation, further supports vasculitic involvement, as seen in our patient. Treatment of SLE-related mesenteric vasculitis relies on aggressive immunosuppression. While low-dose glucocorticoids (40–120 mg/day) may suffice for mild cases, severe or refractory disease often requires high-dose pulse therapy. In this patient, conventional doses (40–120 mg/day) failed to control symptoms, but escalation to 500 mg/day methylprednisolone sodium succinate led to rapid improvement, consistent with reports that severe vasculitis demands prompt high-dose intervention to prevent bowel ischemia or necrosis.

In terms of treatment, our approach aligns with current guidelines for lupus vasculitis: glucocorticoids are recommended for rapid symptom relief, but the medium-to-long-term goal is to minimize the daily dose to ≤5 mg/day of prednisone equivalent (or discontinue them) to avoid irreversible organ damage from long-term use. For acute life-threatening conditions (such as progressive mesenteric vasculitis with worsening ascites), guidelines specifically advocate for high-dose intravenous methylprednisolone (250–1000 mg/day for 3 days)—a strategy that proved effective in our patient, who failed to respond to conventional doses (40–120 mg/day) but improved rapidly after 500 mg/day pulse therapy. Early initiation of immunosuppressants is also advised to reduce glucocorticoid dependence. This is reflected in our patient’s treatment plan, where cyclophosphamide is used in the initial disease control phase.

Compared to existing few case reports on SLE-related mesenteric vasculitis (4–6), which often focus on acute management and short-term outcomes, this report provides valuable data on long-term follow-up (10 months post-discharge), demonstrating sustained disease stability with a combination of conventional immunosuppressants and biologic agents. Such extended follow-up is critical to understanding the role of timely diagnosis, persistence of disease remission, and maintenance therapy in preventing recurrence, in which clinical data is still limited. In terms of limitations, this case lacks the CBC data from the initial onset of urticaria.

The absence of classic SLE cutaneous or joint symptoms in this case underscores the heterogeneity of SLE presentations. Gastrointestinal involvement may be the sole or initial manifestation, and in severe cases, gastrointestinal bleeding (7) or acute abdomen (8) may even occur. Therefore, serological testing and adherence to standardized standards are crucial for diagnosis. Delayed recognition or inadequate treatment, as seen in this patient’s initial course, can lead to progressive intestinal damage, ascites, and multi-organ failure, highlighting the critical role of timely, guideline-driven intervention.

## Conclusions

LMV requires a multidisciplinary approach, combining clinical vigilance, radiological findings, serological testing and alignment with standardized diagnostic criteria (such as the 2019 EULAR/ACR criteria) for early and accurate diagnosis. Consistent with current lupus vasculitis guidelines, high-dose glucocorticoid pulse therapy may be effective in managing severe cases, emphasizing the importance of aggressive treatment to reduce morbidity and mortality. In addition, the 10 months follow-up data emphasizes the importance of prolonged monitoring and personalized treatment adjustment to achieve sustained remission, offering insights into optimizing long-term outcomes for this rare complication.

## Data Availability

The original contributions presented in the study are included in the article/**Supplementary Material**. Further inquiries can be directed to the corresponding author/s.
