# Supplementary figures and images for: Lupus mesenteric vasculitis disguised as urticaria with abdominal pain: a case report with 10 months follow-up observation
Source: Front Immunol. 2025 Oct 29;16:1679036. doi: 10.3389/fimmu.2025.1679036 (PMC12605244; doi:10.3389/fimmu.2025.1679036)

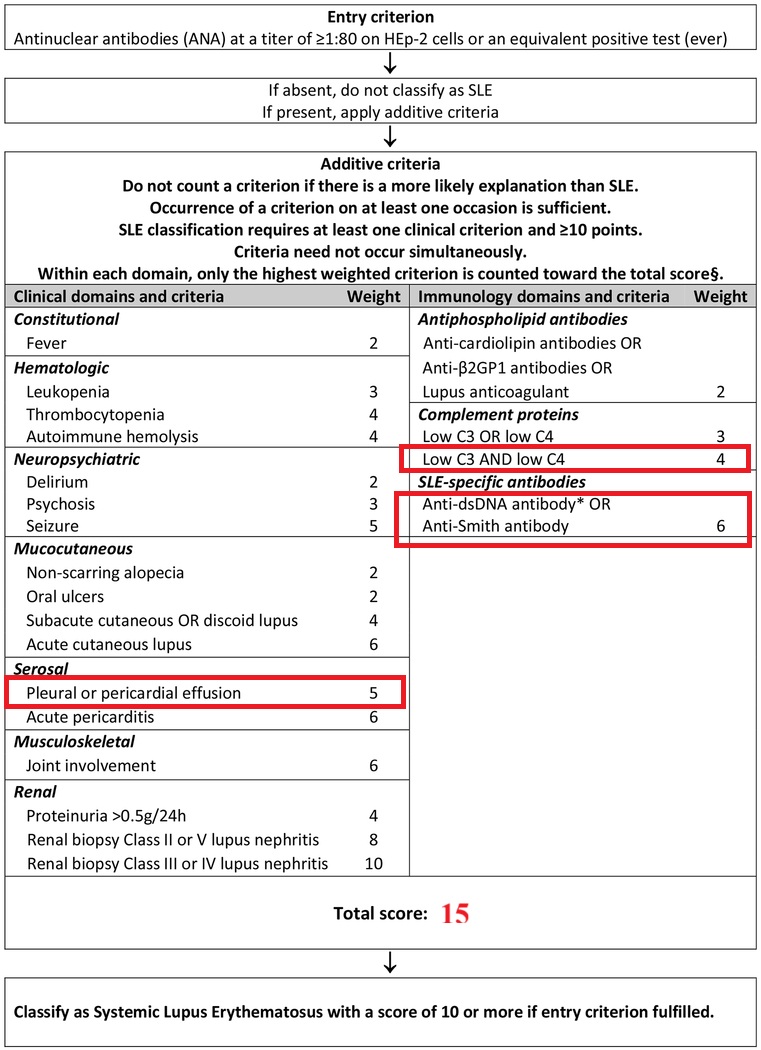

Supplement: Supplementary file 1 [file Image1.jpg]
